# Author Correction: Federated learning enables big data for rare cancer boundary detection

**DOI:** 10.1038/s41467-023-36188-7

**Published:** 2023-01-26

**Authors:** Sarthak Pati, Ujjwal Baid, Brandon Edwards, Micah Sheller, Shih-Han Wang, G. Anthony Reina, Patrick Foley, Alexey Gruzdev, Deepthi Karkada, Christos Davatzikos, Chiharu Sako, Satyam Ghodasara, Michel Bilello, Suyash Mohan, Philipp Vollmuth, Gianluca Brugnara, Chandrakanth J. Preetha, Felix Sahm, Klaus Maier-Hein, Maximilian Zenk, Martin Bendszus, Wolfgang Wick, Evan Calabrese, Jeffrey Rudie, Javier Villanueva-Meyer, Soonmee Cha, Madhura Ingalhalikar, Manali Jadhav, Umang Pandey, Jitender Saini, John Garrett, Matthew Larson, Robert Jeraj, Stuart Currie, Russell Frood, Kavi Fatania, Raymond Y. Huang, Ken Chang, Carmen Balaña, Jaume Capellades, Josep Puig, Johannes Trenkler, Josef Pichler, Georg Necker, Andreas Haunschmidt, Stephan Meckel, Gaurav Shukla, Spencer Liem, Gregory S. Alexander, Joseph Lombardo, Joshua D. Palmer, Adam E. Flanders, Adam P. Dicker, Haris I. Sair, Craig K. Jones, Archana Venkataraman, Meirui Jiang, Tiffany Y. So, Cheng Chen, Pheng Ann Heng, Qi Dou, Michal Kozubek, Filip Lux, Jan Michálek, Petr Matula, Miloš Keřkovský, Tereza Kopřivová, Marek Dostál, Václav Vybíhal, Michael A. Vogelbaum, J. Ross Mitchell, Joaquim Farinhas, Joseph A. Maldjian, Chandan Ganesh Bangalore Yogananda, Marco C. Pinho, Divya Reddy, James Holcomb, Benjamin C. Wagner, Benjamin M. Ellingson, Timothy F. Cloughesy, Catalina Raymond, Talia Oughourlian, Akifumi Hagiwara, Chencai Wang, Minh-Son To, Sargam Bhardwaj, Chee Chong, Marc Agzarian, Alexandre Xavier Falcão, Samuel B. Martins, Bernardo C. A. Teixeira, Flávia Sprenger, David Menotti, Diego R. Lucio, Pamela LaMontagne, Daniel Marcus, Benedikt Wiestler, Florian Kofler, Ivan Ezhov, Marie Metz, Rajan Jain, Matthew Lee, Yvonne W. Lui, Richard McKinley, Johannes Slotboom, Piotr Radojewski, Raphael Meier, Roland Wiest, Derrick Murcia, Eric Fu, Rourke Haas, John Thompson, David Ryan Ormond, Chaitra Badve, Andrew E. Sloan, Vachan Vadmal, Kristin Waite, Rivka R. Colen, Linmin Pei, Murat Ak, Ashok Srinivasan, J. Rajiv Bapuraj, Arvind Rao, Nicholas Wang, Ota Yoshiaki, Toshio Moritani, Sevcan Turk, Joonsang Lee, Snehal Prabhudesai, Fanny Morón, Jacob Mandel, Konstantinos Kamnitsas, Ben Glocker, Luke V. M. Dixon, Matthew Williams, Peter Zampakis, Vasileios Panagiotopoulos, Panagiotis Tsiganos, Sotiris Alexiou, Ilias Haliassos, Evangelia I. Zacharaki, Konstantinos Moustakas, Christina Kalogeropoulou, Dimitrios M. Kardamakis, Yoon Seong Choi, Seung-Koo Lee, Jong Hee Chang, Sung Soo Ahn, Bing Luo, Laila Poisson, Ning Wen, Pallavi Tiwari, Ruchika Verma, Rohan Bareja, Ipsa Yadav, Jonathan Chen, Neeraj Kumar, Marion Smits, Sebastian R. van der Voort, Ahmed Alafandi, Fatih Incekara, Maarten M. J. Wijnenga, Georgios Kapsas, Renske Gahrmann, Joost W. Schouten, Hendrikus J. Dubbink, Arnaud J. P. E. Vincent, Martin J. van den Bent, Pim J. French, Stefan Klein, Yading Yuan, Sonam Sharma, Tzu-Chi Tseng, Saba Adabi, Simone P. Niclou, Olivier Keunen, Ann-Christin Hau, Martin Vallières, David Fortin, Martin Lepage, Bennett Landman, Karthik Ramadass, Kaiwen Xu, Silky Chotai, Lola B. Chambless, Akshitkumar Mistry, Reid C. Thompson, Yuriy Gusev, Krithika Bhuvaneshwar, Anousheh Sayah, Camelia Bencheqroun, Anas Belouali, Subha Madhavan, Thomas C. Booth, Alysha Chelliah, Marc Modat, Haris Shuaib, Carmen Dragos, Aly Abayazeed, Kenneth Kolodziej, Michael Hill, Ahmed Abbassy, Shady Gamal, Mahmoud Mekhaimar, Mohamed Qayati, Mauricio Reyes, Ji Eun Park, Jihye Yun, Ho Sung Kim, Abhishek Mahajan, Mark Muzi, Sean Benson, Regina G. H. Beets-Tan, Jonas Teuwen, Alejandro Herrera-Trujillo, Maria Trujillo, William Escobar, Ana Abello, Jose Bernal, Jhon Gómez, Joseph Choi, Stephen Baek, Yusung Kim, Heba Ismael, Bryan Allen, John M. Buatti, Aikaterini Kotrotsou, Hongwei Li, Tobias Weiss, Michael Weller, Andrea Bink, Bertrand Pouymayou, Hassan F. Shaykh, Joel Saltz, Prateek Prasanna, Sampurna Shrestha, Kartik M. Mani, David Payne, Tahsin Kurc, Enrique Pelaez, Heydy Franco-Maldonado, Francis Loayza, Sebastian Quevedo, Pamela Guevara, Esteban Torche, Cristobal Mendoza, Franco Vera, Elvis Ríos, Eduardo López, Sergio A. Velastin, Godwin Ogbole, Mayowa Soneye, Dotun Oyekunle, Olubunmi Odafe-Oyibotha, Babatunde Osobu, Mustapha Shu’aibu, Adeleye Dorcas, Farouk Dako, Amber L. Simpson, Mohammad Hamghalam, Jacob J. Peoples, Ricky Hu, Anh Tran, Danielle Cutler, Fabio Y. Moraes, Michael A. Boss, James Gimpel, Deepak Kattil Veettil, Kendall Schmidt, Brian Bialecki, Sailaja Marella, Cynthia Price, Lisa Cimino, Charles Apgar, Prashant Shah, Bjoern Menze, Jill S. Barnholtz-Sloan, Jason Martin, Spyridon Bakas

**Affiliations:** 1 Center for Biomedical Image Computing and Analytics (CBICA), University of Pennsylvania, Philadelphia, PA USA; 2 Department of Radiology, Perelman School of Medicine, University of Pennsylvania, Philadelphia, PA USA; 3 Department of Pathology and Laboratory Medicine, Perelman School of Medicine, University of Pennsylvania, Philadelphia, PA USA; 4 Department of Informatics, Technical University of Munich, Munich, Bavaria Germany; 5 Intel Corporation, Santa Clara, CA USA; 6 Department of Neuroradiology, Heidelberg University Hospital, Heidelberg, Germany; 7 Clinical Cooperation Unit Neuropathology, German Cancer Consortium (DKTK) within the German Cancer Research Center (DKFZ), Heidelberg, Germany; 8 Department of Neuropathology, Heidelberg University Hospital, Heidelberg, Germany; 9 Division of Medical Image Computing, German Cancer Research Center, Heidelberg, Germany; 10 Pattern Analysis and Learning Group, Department of Radiation Oncology, Heidelberg University Hospital, Heidelberg, Germany; 11 Neurology Clinic, Heidelberg University Hospital, Heidelberg, Germany; 12 Department of Radiology & Biomedical Imaging, University of California San Francisco, San Francisco, CA USA; 13 Symbiosis Center for Medical Image Analysis, Symbiosis International University, Pune, Maharashtra India; 14 Department of Neuroimaging and Interventional Radiology, National Institute of Mental Health and Neurosciences, Bangalore, Karnataka India; 15 Department of Radiology, School of Medicine and Public Health, University of Wisconsin, Madison, WI USA; 16 Department of Medical Physics, School of Medicine and Public Health, University of Wisconsin, Madison, WI USA; 17 Leeds Teaching Hospitals Trust, Department of Radiology, Leeds, UK; 18 Department of Radiology, Brigham and Women’s Hospital, Harvard Medical School, Boston, MA USA; 19 Athinoula A. Martinos Center for Biomedical Imaging, Massachusetts General Hospital, Charlestown, MA USA; 20 Catalan Institute of Oncology, Badalona, Spain; 21 Consorci MAR Parc de Salut de Barcelona, Catalonia, Spain; 22 Department of Radiology (IDI), Girona Biomedical Research Institute (IdIBGi), Josep Trueta University Hospital, Girona, Spain; 23 Institute of Neuroradiology, Neuromed Campus (NMC), Kepler University Hospital Linz, Linz, Austria; 24 Department of Neurooncology, Neuromed Campus (NMC), Kepler University Hospital Linz, Linz, Austria; 25 Institute of Diagnostic and Interventional Neuroradiology, RKH Klinikum Ludwigsburg, Ludwigsburg, Germany; 26 Department of Radiation Oncology, Christiana Care Health System, Philadelphia, PA USA; 27 Sidney Kimmel Medical College, Thomas Jefferson University, Philadelphia, PA USA; 28 Department of Radiation Oncology, University of Maryland, Baltimore, MD USA; 29 Department of Radiation Oncology, Sidney Kimmel Cancer Center, Thomas Jefferson University, Philadelphia, PA USA; 30 Department of Radiation Oncology, The James Cancer Hospital and Solove Research Institute, The Ohio State University Comprehensive Cancer Center, Columbus, OH USA; 31 Department of Radiology, Sidney Kimmel Cancer Center, Thomas Jefferson University, Philadelphia, PA USA; 32 The Russell H. Morgan Department of Radiology and Radiological Science, Johns Hopkins University School of Medicine, Baltimore, MD USA; 33 The Malone Center for Engineering in Healthcare, The Whiting School of Engineering, Johns Hopkins University, Baltimore, MD USA; 34 Department of Electrical and Computer Engineering, Whiting School of Engineering, Johns Hopkins University, Baltimore, MD USA; 35 The Chinese University of Hong Kong, Hong Kong, China; 36 Centre for Biomedical Image Analysis, Faculty of Informatics, Masaryk University, Brno, Czech Republic; 37 Department of Radiology and Nuclear Medicine, Faculty of Medicine, Masaryk University, Brno and University Hospital Brno, Brno, Czech Republic; 38 Department of Biophysics, Faculty of Medicine, Masaryk University, Brno, Czech Republic; 39 Department of Neurosurgery, Faculty of Medicine, Masaryk University, Brno, and University Hospital and Czech Republic, Brno, Czech Republic; 40 Department of Neuro Oncology, H. Lee Moffitt Cancer Center and Research Institute, Tampa, FL USA; 41 University of Alberta, Edmonton, AB Canada; 42 Alberta Machine Intelligence Institute, Edmonton, AB Canada; 43 Department of Radiology, H. Lee Moffitt Cancer Center and Research Institute, Tampa, FL USA; 44 University of Texas Southwestern Medical Center, Dallas, TX USA; 45 UCLA Brain Tumor Imaging Laboratory (BTIL), Center for Computer Vision and Imaging Biomarkers, Department of Radiological Sciences, David Geffen School of Medicine, University of California Los Angeles, Los Angeles, CA USA; 46 UCLA Neuro-Oncology Program, Department of Neurology, David Geffen School of Medicine, University of California Los Angeles, Los Angeles, CaA USA; 47 Department of Radiological Sciences, David Geffen School of Medicine, University of California Los Angeles, Los Angeles, CA USA; 48 College of Medicine and Public Health, Flinders University, Bedford Park, SA Australia; 49 Division of Surgery and Perioperative Medicine, Flinders Medical Centre, Bedford Park, SA Australia; 50 South Australia Medical Imaging, Flinders Medical Centre, Bedford Park, SA Australia; 51 Department of Neurology, Baylor College of Medicine, Houston, TX USA; 52 Institute of Computing, University of Campinas, Campinas, São Paulo Brazil; 53 Federal Institute of São Paulo, Campinas, São Paulo Brazil; 54 Instituto de Neurologia de Curitiba, Curitiba, Paraná Brazil; 55 Department of Radiology, Hospital de Clínicas da Universidade Federal do Paraná, Curitiba, Paraná, Brazil; 56 Department of Informatics, Universidade Federal do Paraná, Curitiba, Paraná, Brazil; 57 Department of Radiology, Washington University in St. Louis, St. Louis, MO USA; 58 Department of Diagnostic and Interventional Neuroradiology, School of Medicine, Klinikum rechts der Isar, Technical University of Munich, Munich, Germany; 59 TranslaTUM (Zentralinstitut für translationale Krebsforschung der Technischen Universität München), Klinikum rechts der Isar, Munich, Germany; 60 Image-Based Biomedical Modeling, Department of Informatics, Technical University of Munich, Munich, Germany; 61 Department of Radiology, NYU Grossman School of Medicine, New York, NY USA; 62 Department of Neurosurgery, NYU Grossman School of Medicine, New York, NY USA; 63 Support Center for Advanced Neuroimaging, University Institute of Diagnostic and Interventional Neuroradiology, University Hospital Bern, Inselspital, University of Bern, Bern, Switzerland; 64 Department of Neurosurgery, Anschutz Medical Campus, University of Colorado, Aurora, CO USA; 65 Department of Radiology, University Hospitals Cleveland, Cleveland, OH USA; 66 Department of Neurological Surgery, University Hospitals-Seidman Cancer Center, Cleveland, OH USA; 67 Case Comprehensive Cancer Center, Cleveland, OH USA; 68 Department of Neurosurgery, Case Western Reserve University School of Medicine, Cleveland, OH USA; 69 National Cancer Institute, National Institute of Health, Division of Cancer Epidemiology and Genetics, Bethesda, MD USA; 70 Department of Radiology, Neuroradiology Division, University of Pittsburgh, Pittsburgh, PA USA; 71 Department of Diagnostic Radiology, University of Texas MD Anderson Cancer Center, Houston, TX USA; 72 University of Pittsburgh Medical Center, Pittsburgh, PA USA; 73 Department of Neuroradiology, University of Michigan, Ann Arbor, MI USA; 74 Department of Computational Medicine and Bioinformatics, University of Michigan, Ann Arbor, MI USA; 75 Department of Radiology, Baylor College of Medicine, Houston, TX USA; 76 Department of Computing, Imperial College London, London, UK; 77 Institute of Biomedical Engineering, Department of Engineering Science, University of Oxford, Oxford, UK; 78 Department of Radiology, Imperial College NHS Healthcare Trust, London, UK; 79 Computational Oncology Group, Institute for Global Health Innovation, Imperial College London, London, UK; 80 Department of NeuroRadiology, University of Patras, Patras, Greece; 81 Department of Neurosurgery, University of Patras, Patras, Greece; 82 Clinical Radiology Laboratory, Department of Medicine, University of Patras, Patras, Greece; 83 Department of Electrical and Computer Engineering, University of Patras, Patras, Greece; 84 Department of Neuro-Oncology, University of Patras, Patras, Greece; 85 Department of Radiation Oncology, University of Patras, Patras, Greece; 86 Yonsei University College of Medicine, Seoul, Korea; 87 Department of Radiation Oncology, Henry Ford Health System, Detroit, MI USA; 88 Public Health Sciences, Henry Ford Health System, Detroit, MI USA; 89 SJTU-Ruijin-UIH Institute for Medical Imaging Technology, Ruijin Hospital, Shanghai Jiao Tong University School of Medicine, 200025 Shanghai, China; 90 Case Western Reserve University, Cleveland, OH USA; 91 Department of Radiology and Nuclear Medicine, Erasmus MC University Medical Centre Rotterdam, Rotterdam, Netherlands; 92 Department of Neurosurgery, Brain Tumor Center, Erasmus MC University Medical Centre Rotterdam, Rotterdam, Netherlands; 93 Department of Neurology, Brain Tumor Center, Erasmus MC Cancer Institute, Rotterdam, Netherlands; 94 Department of Pathology, Brain Tumor Center, Erasmus MC Cancer Institute, Rotterdam, Netherlands; 95 Biomedical Imaging Group Rotterdam, Department of Radiology and Nuclear Medicine, Erasmus MC University Medical Centre Rotterdam, Rotterdam, Netherlands; 96 Department of Radiation Oncology, Icahn School of Medicine at Mount Sinai, New York, NY USA; 97 NORLUX Neuro-Oncology Laboratory, Department of Cancer Research, Luxembourg Institute of Health, Luxembourg, Luxembourg; 98 Translation Radiomics, Department of Cancer Research, Luxembourg Institute of Health, Luxembourg, Luxembourg; 99 Luxembourg Center of Neuropathology, Laboratoire National De Santé, Luxembourg, Luxembourg; 100 Department of Computer Science, Université de Sherbrooke, Sherbrooke, QC Canada; 101 Centre de Recherche du Centre Hospitalière Universitaire de Sherbrooke, Sherbrooke, QC Canada; 102 Division of Neurosurgery and Neuro-Oncology, Faculty of Medicine and Health Science, Université de Sherbrooke, Sherbrooke, QC Canada; 103 Department of Nuclear Medicine and Radiobiology, Sherbrooke Molecular Imaging Centre, Université de Sherbrooke, Sherbrooke, QC Canada; 104 Electrical and Computer Engineering, Vanderbilt University, Nashville, TN USA; 105 Department of Computer Science, Vanderbilt University, Nashville, TN USA; 106 Department of Neurosurgery, Vanderbilt University Medical Center, Nashville, TN USA; 107 Innovation Center for Biomedical Informatics (ICBI), Georgetown University, Washington, DC USA; 108 Division of Neuroradiology & Neurointerventional Radiology, Department of Radiology, MedStar Georgetown University Hospital, Washington, DC USA; 109 School of Biomedical Engineering & Imaging Sciences, King’s College London, London, UK; 110 Department of Neuroradiology, Ruskin Wing, King’s College Hospital NHS Foundation Trust, London, UK; 111 Stoke Mandeville Hospital, Mandeville Road, Aylesbury, UK; 112 Department of Biomedical and Molecular Sciences, Queen’s University, Kingston, ON Canada; 113 Neosoma Inc., Groton, MA USA; 114 University of Cairo School of Medicine, Giza, Egypt; 115 University of Bern, Bern, Switzerland; 116 Department of Radiology, Asan Medical Center, Seoul, South Korea; 117 The Clatterbridge Cancer Centre NHS Foundation Trust Pembroke Place, Liverpool, UK; 118 Department of Radiology, University of Washington, Seattle, WA USA; 119 Netherlands Cancer Institute, Amsterdam, Netherlands; 120 Department of Radiology, Netherlands Cancer Institute, Amsterdam, Netherlands; 121 GROW School of Oncology and Developmental Biology, Maastricht, Netherlands; 122 Clínica Imbanaco Grupo Quirón Salud, Cali, Colombia; 123 Universidad del Valle, Cali, Colombia; 124 The University of Edinburgh, Edinburgh, UK; 125 Department of Industrial and Systems Engineering, University of Iowa, Iowa, USA; 126 Department of Industrial and Systems Engineering, Department of Radiation Oncology, University of Iowa, Iowa City, IA USA; 127 Department of Radiation Oncology, University of Iowa, Iowa City, IA USA; 128 MD Anderson Cancer Center, University of Texas, Houston, TX USA; 129 Department of Quantitative Biomedicine, University of Zurich, Zurich, Switzerland; 130 Department of Neurology, Clinical Neuroscience Center, University Hospital Zurich and University of Zurich, Zurich, Switzerland; 131 Department of Neuroradiology, Clinical Neuroscience Center, University Hospital Zurich and University of Zurich, Zurich, Switzerland; 132 University of Alabama in Birmingham, Birmingham, AL USA; 133 Department of Biomedical Informatics, Stony Brook University, Stony Brook, New York, USA; 134 Department of Radiation Oncology, Stony Brook University, Stony Brook, NY USA; 135 Department of Radiology, Stony Brook University, Stony Brook, NY USA; 136 Scientific Data Group, Oak Ridge National Laboratory, Oak Ridge, TN USA; 137 Escuela Superior Politecnica del Litoral, Guayaquil, Guayas Ecuador; 138 Sociedad de Lucha Contral el Cancer - SOLCA, Guayaquil Ecuador, Guayaquil, Ecuador; 139 Universidad Católica de Cuenca, Cuenca, Ecuador; 140 Universidad de Concepción, Concepción, Biobío Chile; 141 School of Electronic Engineering and Computer Science, Queen Mary University of London, London, UK; 142 Department of Radiology, University College Hospital Ibadan, Oyo, Nigeria; 143 Clinix Healthcare, Lagos, Lagos, Nigeria; 144 Department of Radiology, Muhammad Abdullahi Wase Teaching Hospital, Kano, Nigeria; 145 Department of Radiology, Obafemi Awolowo University Ile-Ife, Ile-Ife, Osun Nigeria; 146 Center for Global Health, Perelman School of Medicine, University of Pennsylvania, Philadelphia, PA USA; 147 School of Computing, Queen’s University, Kingston, ON Canada; 148 Department of Electrical Engineering, Qazvin Branch, Islamic Azad University, Qazvin, Iran; 149 The Faculty of Arts & Sciences, Queen’s University, Kingston, ON Canada; 150 Department of Oncology, Queen’s University, Kingston, ON Canada; 151 Center for Research and Innovation, American College of Radiology, Philadelphia, PA USA; 152 Data Science Institute, American College of Radiology, Reston, VA USA; 153 Center for Biomedical Informatics and Information Technology, National Cancer Institute (NCI), National Institute of Health, Bethesda, MD USA

**Keywords:** CNS cancer, Computer science, Medical imaging, Medical research, Biomedical engineering

Correction to: *Nature Communications* 10.1038/s41467-022-33407-5, published online 05 December 2022

In this article the author name Carmen Balaña was incorrectly written as Carmen Balaña Quintero. The original article has been corrected.

