# Supplementary material for: Federated learning enables big data for rare cancer boundary detection
Source: Nat Commun. 2022 Dec 5;13:7346. doi: 10.1038/s41467-022-33407-5 (PMC9722782; doi:10.1038/s41467-022-33407-5)
Supplement: Supplementary file 1 — Reporting Summary [file 41467_2022_33407_MOESM1_ESM.pdf]

nature research

Corresponding author(s): Spyridon Bakas, Ph.D

Last updated by author(s): Sep 5, 2022

Reporting Summary

Nature Research wishes to improve the reproducibility of the work that we publish. This form provides structure for consistency and transparency in reporting. For further information on Nature Research policies, see our Editorial Policies and the Editorial Policy Checklist.

Statistics

For all statistical analyses, confirm that the following items are present in the figure legend, table legend, main text, or Methods section.

n/a	Confirmed	
☐	☒	The exact sample size (n) for each experimental group/condition, given as a discrete number and unit of measurement
☐	☒	A statement on whether measurements were taken from distinct samples or whether the same sample was measured repeatedly
☐	☒	The statistical test(s) used AND whether they are one- or two-sided<br>*Only common tests should be described solely by name; describe more complex techniques in the Methods section.*
☒	☐	A description of all covariates tested
☒	☐	A description of any assumptions or corrections, such as tests of normality and adjustment for multiple comparisons
☐	☒	A full description of the statistical parameters including central tendency (e.g. means) or other basic estimates (e.g. regression coefficient) AND variation (e.g. standard deviation) or associated estimates of uncertainty (e.g. confidence intervals)
☐	☒	For null hypothesis testing, the test statistic (e.g. F, t, r) with confidence intervals, effect sizes, degrees of freedom and P value noted<br>*Give P values as exact values whenever suitable.*
☒	☐	For Bayesian analysis, information on the choice of priors and Markov chain Monte Carlo settings
☒	☐	For hierarchical and complex designs, identification of the appropriate level for tests and full reporting of outcomes
☒	☐	Estimates of effect sizes (e.g. Cohen's d, Pearson's r), indicating how they were calculated

Our web collection on statistics for biologists contains articles on many of the points above.

Software and code

Policy information about availability of computer code

Data collection	The data used in this project were collected at the radiology department of each collaborating institution, by directly accessing their local PACS. No additional software was used for data collection. The information of the exact PACS vendor was not provided by each site.
Data analysis	All data analysis of the extracted scans were done using the FeTS Tool (v.0.0.7). The source code of this package and all of its dependencies are open-sourced in https://github.com/FETS-AI/Front-End. We have also made installers available for Ubuntu and CentOS based Linux distributions in https://github.com/FETS-AI/Front-End/releases, as well as containers based on Docker and Singularity. All information related to the data and figures is present in https://github.com/FETS-AI/2022_Manuscript_Supplement.

For manuscripts utilizing custom algorithms or software that are central to the research but not yet described in published literature, software must be made available to editors and reviewers. We strongly encourage code deposition in a community repository (e.g. GitHub). See the Nature Research guidelines for submitting code & software for further information.

Data

Policy information about availability of data

All manuscripts must include a data availability statement. This statement should provide the following information, where applicable:

- Accession codes, unique identifiers, or web links for publicly available datasets
- A list of figures that have associated raw data
- A description of any restrictions on data availability

The datasets used in this study, from the 71 participating sites, are not made publicly available as a collective data collection due to restrictions imposed by acquiring institutions. The public initial model data from 16 sites are publicly available through the BraTS challenge, available from https://www.med.upenn.edu/cbica/brats2020. The data from each of the 55 collaborating sites were neither publicly available during the execution of the study, nor shared among collaborating sites or with the aggregator. They were instead used locally, within each of the acquiring sites, for the training and validation of the global consensus model at each

federated round. The anatomical template used for co-registration during preprocessing is the SRI24 atlas and is available from (https://www.nitrc.org/projects/sri24).

We provide the raw data, the associated python scripts, and specific instructions to reproduce the plots of this study in a GitHub repository, at: https://github.com/FETS-AI/2022_Manuscript_Supplement. The file "SourceData.tgz", in the top directory holds an archive of csv files representing the source data. The python scripts are provided in the 'scripts' folder which utilize these source data and save ".png" images to disc and/or print latex code (for tables) to stdout. Furthermore, we have provided 3 sample validation cases, from the publicly available BraTS dataset, to qualitatively showcase the segmentation differences (small, moderate and large) across the final global consensus model, the public initial model, and the ground truth annotations in the same GitHub repository.

Field-specific reporting

Please select the one below that is the best fit for your research. If you are not sure, read the appropriate sections before making your selection.

- [x] Life sciences
- [ ] Behavioural & social sciences
- [ ] Ecological, evolutionary & environmental sciences

For a reference copy of the document with all sections, see nature.com/documents/nr-reporting-summary-flat.pdf

Life sciences study design

All studies must disclose on these points even when the disclosure is negative.

Sample size	The public initial model was used to initialize the FL training using 231 cases from 16 international sites from the BraTS Challenge, with varying contributing cases across sites. The eligibility of collaborating sites to participate in the federation was determined based on data availability, and approval by their respective institutional review board. 55 sites participated as independent collaborators in the study defining a dataset of 6,083 cases. From these 55 sites, 49 were chosen to be part of the training phase, and the remaining 6 were categorized as "out-of-sample". No statistical methods were used to predetermine the sample size. The number of cases used in this study are the largest to-date for any glioblastoma delineation study and showcases the applicability of federated learning in a large-scale setting.
Data exclusions	The data required for this study required displaying the radiological features of glioblastoma scanned with multi-parametric MRI to characterize the anatomical tissue structure. Each case is specifically described by i) native T1-weighted (T1), ii) Gadolinium-enhanced T1-weighted (T1Gd), iii) T2-weighted (T2), and iv) T2-weighted-Fluid-Attenuated-Inversion-Recovery (T2-FLAIR) MRI scans. Cases with any of these sequences missing were not included in the study. Note that no inclusion/exclusion criterion applied relating to the type of acquisition (i.e., both 2D axial and 3D acquisitions were included, with a preference for 3D if available), or the exact type of sequence (e.g., MP-RAGE vs SPGR). The only exclusion criterion was for T1-FLAIR scans that were intentionally excluded to avoid mixing varying tissue appearance due to the type of sequence, across native T1-weighted scans.
Replication	We have performed this experiment in 4 distinct stages: simulation within a single node, simulation within the same network, a small federation with communication between different machines across different networks, and a full federation with all sites. Apart from the last 2 steps, which used data from external institutions, we evaluated the performance of the resultant model federated with one generated in a centralized manner and found them to be comparable.
Randomization	The data was randomized before getting passed to the network as training, validation, and out-of-sample hold-out cases. During each experiment, these splits were preserved to prevent any data leakage.
Blinding	Federated learning allows training a model across multiple collaborators where none of them have any access to the other's data. This results in a nature blinding to the data acquisition and allocation.

Reporting for specific materials, systems and methods

We require information from authors about some types of materials, experimental systems and methods used in many studies. Here, indicate whether each material, system or method listed is relevant to your study. If you are not sure if a list item applies to your research, read the appropriate section before selecting a response.

Materials & experimental systems

n/a	Involved in the study	
x		Antibodies
x		Eukaryotic cell lines
x		Palaeontology and archaeology
		Animals and other organisms
	x	Human research participants
x		Clinical data
x		Dual use research of concern

Methods

n/a	Involved in the study	
x		ChIP-seq
x		Flow cytometry
	x	MRI-based neuroimaging

Animals and other organisms

Policy information about studies involving animals; ARRIVE guidelines recommended for reporting animal research

Laboratory animals	N.A.
Wild animals	N.A.
Field-collected samples	N.A.
Ethics oversight	N.A.

Note that full information on the approval of the study protocol must also be provided in the manuscript.

Human research participants

Policy information about studies involving human research participants

Population characteristics	The MRI scanners used for data acquisition were from multiple vendors (i.e., Siemens, GE, Philips, Hitachi, Toshiba), with magnetic field strength ranging from 1T to 3T. The data from all 55 collaborating sites followed a male:female ratio of 1.47:1 with age ranging between 7 and 94 years.
Recruitment	The data used in this study was acquired retrospectively.
Ethics oversight	*Identify the organization(s) that approved the study protocol.*

Note that full information on the approval of the study protocol must also be provided in the manuscript.

Magnetic resonance imaging

Experimental design

Design type	*Indicate task or resting state; event-related or block design.*
Design specifications	*Specify the number of blocks, trials or experimental units per session and/or subject, and specify the length of each trial or block (if trials are blocked) and interval between trials.*
Behavioral performance measures	*State number and/or type of variables recorded (e.g. correct button press, response time) and what statistics were used to establish that the subjects were performing the task as expected (e.g. mean, range, and/or standard deviation across subjects).*

Acquisition

Imaging type(s)	Structural
Field strength	1.5T - 3T
Sequence & imaging parameters	i) native T1-weighted (T1), ii) Gadolinium-enhanced T1-weighted (T1Gd), iii) T2-weighted (T2), and iv) T2-weighted-Fluid-Attenuated-Inversion-Recovery (T2-FLAIR)
Area of acquisition	The entire brain was scanned.

Diffusion MRI ☐ Used ☒ Not used

Preprocessing

Preprocessing software	We performed DICOM to NIfTI conversion, co-registration to anatomical template, and brain extraction as described for the BraTS Challenge. All the tools used for this study are open-sourced in https://github.com/FETS-AI/Front-End.
Normalization	We performed z-scoring normalization on the non-zero areas of the extracted brain. All the tools used for this study are open-sourced in https://github.com/FETS-AI/Front-End.
Normalization template	No normalization template was used.
Noise and artifact removal	We did not perform any noise or artifact removal from the data to preserve maximal fidelity.
Volume censoring	We did not perform any volume censoring.

Statistical modeling & inference

Model type and settings	Wilcoxon two-sided signed-rank test

Effect(s) tested	Significance of differences of the generated predictions.

Specify type of analysis: ☐ Whole brain ☒ ROI-based ☐ Both

Anatomical location(s)	Different tumor locations: whole tumor, tumor core, and the enhancing part of the tumor.
Statistic type for inference (See Eklund et al. 2016)	*Specify voxel-wise or cluster-wise and report all relevant parameters for cluster-wise methods.*
Correction	*Describe the type of correction and how it is obtained for multiple comparisons (e.g. FWE, FDR, permutation or Monte Carlo).*

Models & analysis

n/a	Involved in the study
☒	☐ Functional and/or effective connectivity
☒	☐ Graph analysis
☒	☐ Multivariate modeling or predictive analysis
