# Supplementary material for: Federated learning enables big data for rare cancer boundary detection
Source: Nat Commun. 2022 Dec 5;13:7346. doi: 10.1038/s41467-022-33407-5 (PMC9722782; doi:10.1038/s41467-022-33407-5)
Supplement: Supplementary file 3 — Supplementary Figures and Tables [file 41467_2022_33407_MOESM3_ESM.pdf]

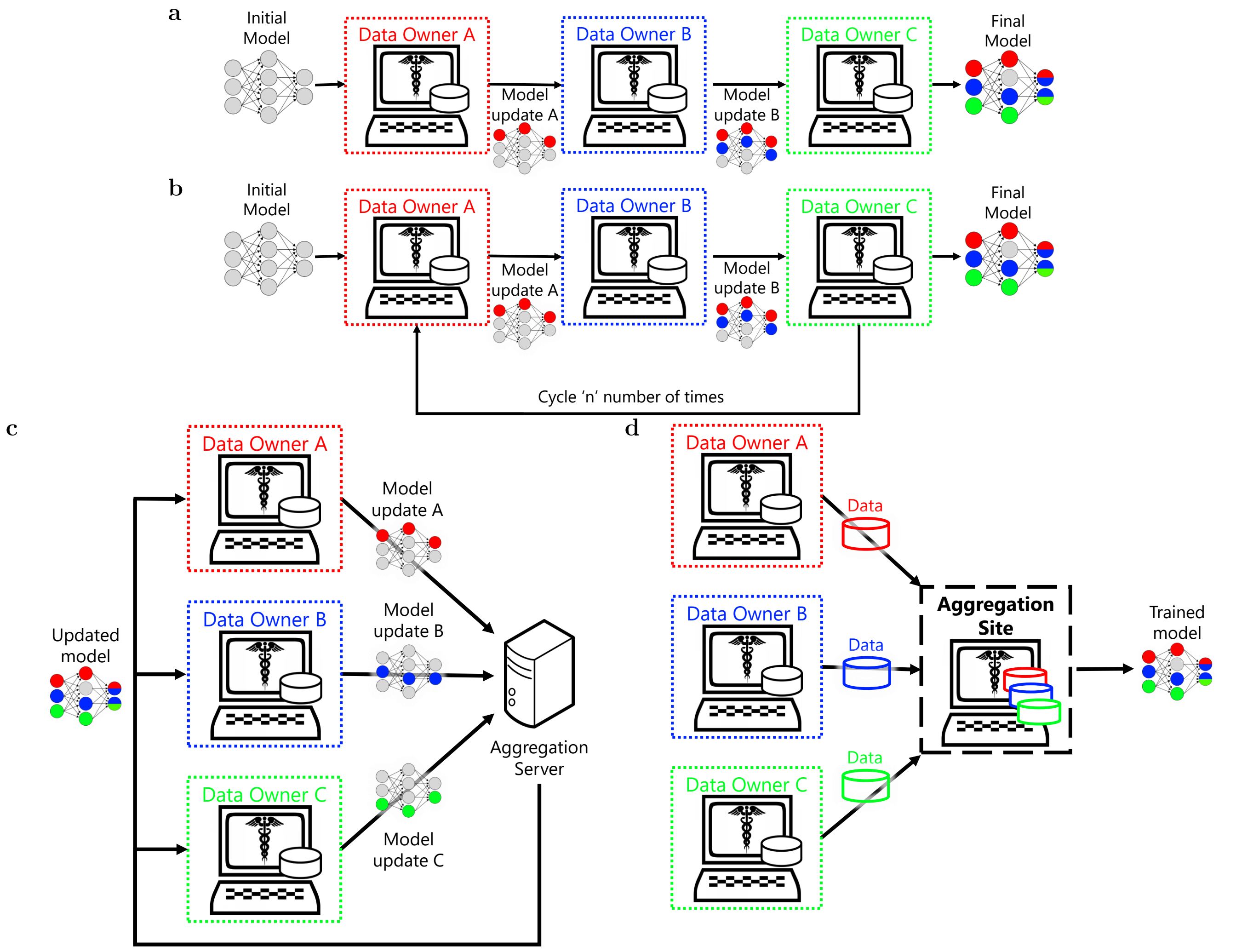


Suppl Figure 1: Illustration of the different approaches to train DL models during multi-site collaborations. **a**, Institutional incremental learning (IIL). **b**, Cyclic Institutional incremental learning. **c**, Federated learning by independent aggregation. **d**, Centralized learning by aggregating data.

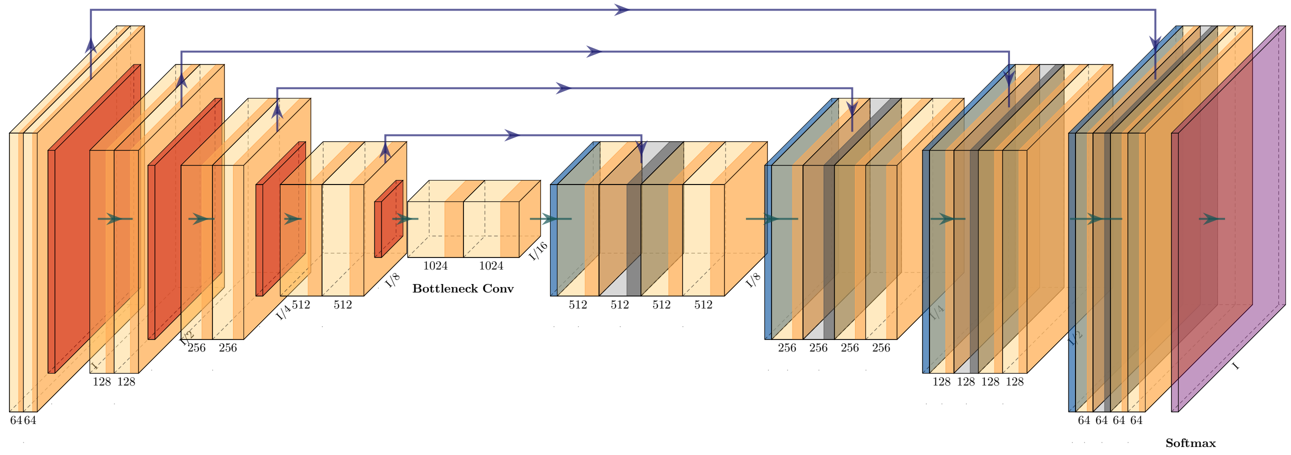


Suppl Figure 2: Illustration of the U-Net architecture with residual connections, plotted using PlotNeuralNet github.com/HarisIqbal88/PlotNeuralNet. One spatial dimension is left out for clarity.

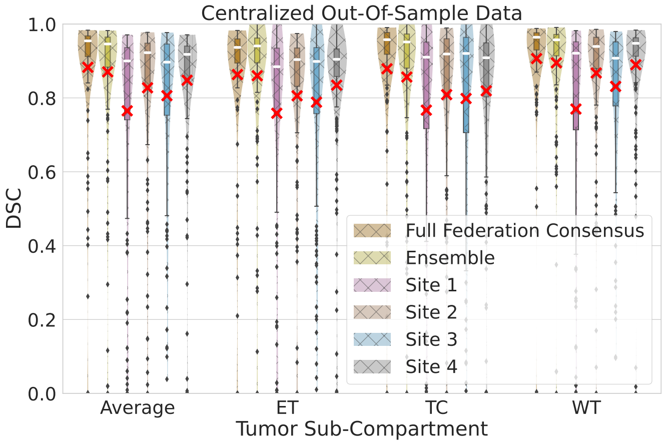


Suppl Figure 3: Comparative performance evaluation of the final consensus model against the centralized out of sample data ($n = 154$ biologically independent cases), per tumor sub-compartment and averaged across cases. Comparison with the public initial model with each single site model (for collaborators holding $> 200$ samples), as well as with their ensemble. Significance of results (Wilcoxon two-sided signed-rank test): Site 1: $p_{Average} = 2 \times 10^{-7}$, $p_{ET} = 4 \times 10^{-8}$, $p_{TC} = 6 \times 10^{-8}$, $p_{WT} = 2 \times 10^{-9}$; Site 2: $p_{Average} = 5 \times 10^{-6}$, $p_{ET} = 1 \times 10^{-5}$, $p_{TC} = 1 \times 10^{-4}$, $p_{WT} = 1 \times 10^{-4}$, Site 3: $p_{Average} = 3 \times 10^{-7}$, $p_{ET} = 2 \times 10^{-6}$, $p_{TC} = 1 \times 10^{-6}$, $p_{WT} = 6 \times 10^{-9}$, Site 4: $p_{Average} = 4 \times 10^{-6}$, $p_{ET} = 5 \times 10^{-5}$, $p_{TC} = 8 \times 10^{-7}$, $p_{WT} = 8 \times 10^{-3}$. Note the box and whiskers inside each violin plot, represent the true min and max values. The top and bottom of each "box" depict the 3rd and 1st quartile of each measure. The white line and the red '×', within each box, indicate the median and mean values, respectively. The fact that these are not necessarily at the centre of each box indicates the skewness of the distribution over different cases. The "whiskers" drawn above and below each box depict the extremal observations still within 1.5 times the interquartile range, above the 3rd or below the 1st quartile.

Table 1: Average Dice Similarity Coefficient ($DSC$) over cases for various *singlet* and *triplet* models against the centralized out-of-sample data for various regions (ET: enhancing tumor, TC: tumor core, WT: whole tumor).

| **Model** | | $DSC$ | | | |
|---|---|---|---|---|---|
| **Type** | **Index** | **Average** | **ET** | **TC** | **WT** |
| ***singlet*** | 0 | 0.743996 | 0.683714 | 0.713348 | 0.834925 |
| | 1 | 0.745362 | 0.690743 | 0.714308 | 0.831035 |
| | 2 | 0.742246 | 0.6806 | 0.711266 | 0.834871 |
| | 3 | 0.745255 | 0.697808 | 0.710747 | 0.82721 |
| | 4 | 0.74213 | 0.687588 | 0.711013 | 0.827789 |
| ***triplet*** | 0 | 0.749014 | 0.697808 | 0.714308 | 0.834925 |
| | 1 | 0.750356 | 0.697808 | 0.718336 | 0.834925 |
| | 2 | 0.748694 | 0.697808 | 0.713348 | 0.834925 |
| | 3 | 0.748996 | 0.697808 | 0.714308 | 0.834871 |
| | 4 | 0.746683 | 0.697808 | 0.714308 | 0.827934 |

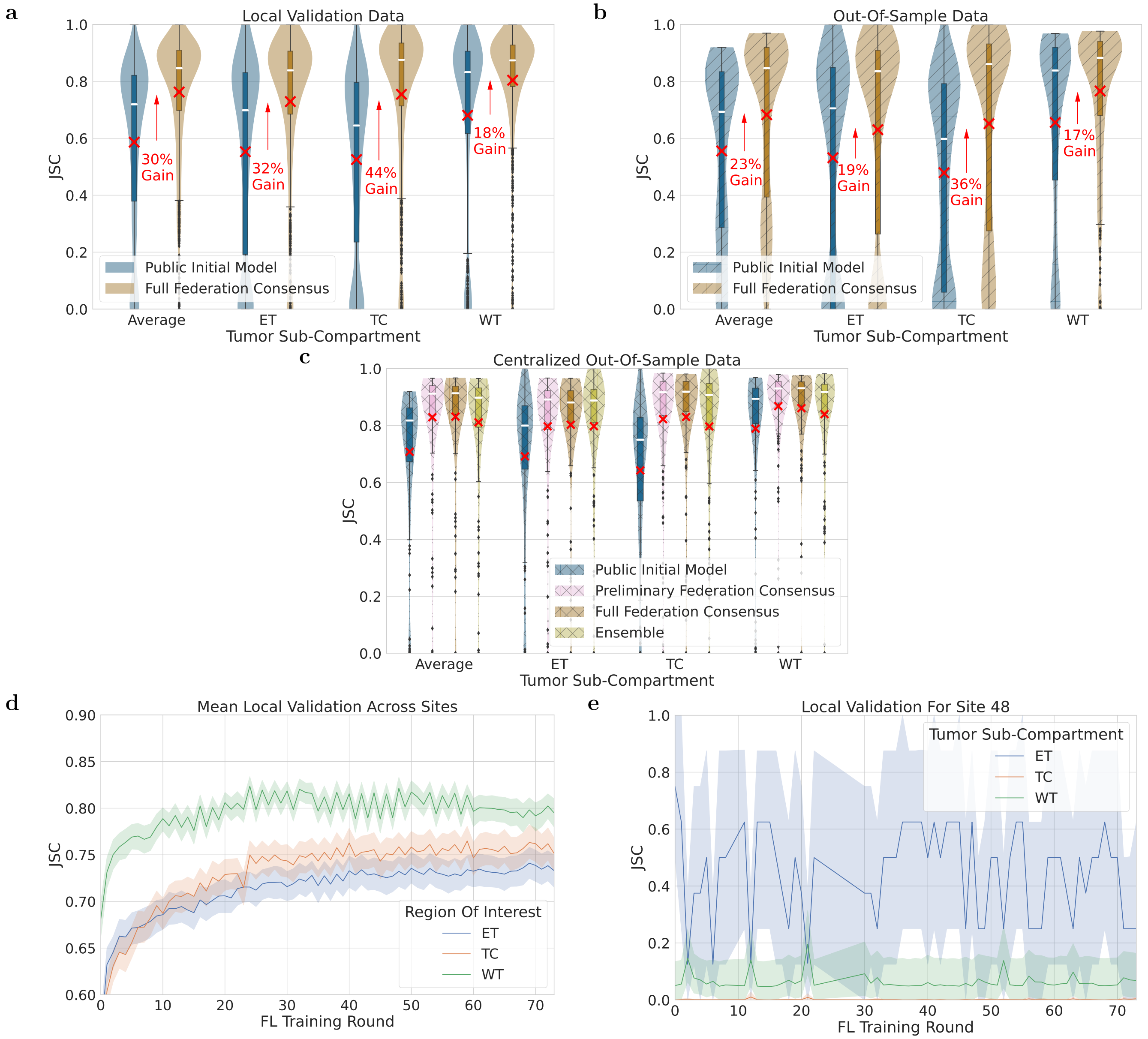


Suppl Figure 4: Illustrations of the equivalent Jaccard Similarity Coefficient ($JSC$) for all the figures in the main text depicting the Dice Similarity Coefficient ($DSC$). **a-b**, $JSC$ for Figure 1(c) and (d). **c**, $JSC$ for Figure 2. **d-e**, $JSC$ for Figure 3(a) and (b).

Table 2: P values providing significance of the difference in mean Dice Similarity Coefficient ($DSC$) scores across cases between each *singlet* and *triplet* model pairing on the centralized out-of-sample data (with $n = 154$ biologically independent cases) per tumor region (ET: enhancing tumor, TC: tumor core, WT: whole tumor). Significance is calculated using Wilcoxon two-sided signed-ranked test where sample pairs are correspondingly the *singlet* and *triplet* model score for a single case. Entries with 'NA' correspond to scoring where the *singlet* model was the member of the *triplet* responsible for producing the particular tumor sub-compartment output, so that the scores between the *singlet* and the *triplet* model for that tumor sub-compartment were identical.

| | | ***singlet* Index** | | | | |
|---|---|---|---|---|---|---|
| ***triplet* Index** | **metric** | **0** | **1** | **2** | **3** | 4 |
| 0 | Average | $5.9 \times 10^{-6}$ | 0.31 | $8.3 \times 10^{-12}$ | 0.21 | $1.8 \times 10^{-6}$ |
| | ET | $3.2 \times 10^{-19}$ | $5.1 \times 10^{-6}$ | $3.7 \times 10^{-17}$ | NA | $2.3 \times 10^{-22}$ |
| | TC | 0.35 | NA | $2.3 \times 10^{-6}$ | 0.37 | 0.76 |
| | WT | NA | 0.34 | 0.026 | 0.61 | 0.00037 |
| 1 | Average | $5.5 \times 10^{-11}$ | 0.0047 | $1.6 \times 10^{-16}$ | 0.0017 | $1.1 \times 10^{-13}$ |
| | ET | $3.2 \times 10^{-19}$ | $5.1 \times 10^{-6}$ | $3.7 \times 10^{-17}$ | NA | $2.3 \times 10^{-22}$ |
| | TC | 0.012 | 0.013 | $2.4 \times 10^{-9}$ | 0.027 | 0.016 |
| | WT | NA | 0.34 | 0.026 | 0.61 | 0.00037 |
| 2 | Average | $4.3 \times 10^{-19}$ | 0.97 | $7.0 \times 10^{-13}$ | 0.21 | $6.4 \times 10^{-6}$ |
| | ET | $3.2 \times 10^{-19}$ | $5.1 \times 10^{-6}$ | $3.7 \times 10^{-17}$ | NA | $2.3 \times 10^{-22}$ |
| | TC | NA | 0.35 | $2.6 \times 10^{-8}$ | 0.83 | 0.29 |
| | WT | NA | 0.34 | 0.026 | 0.61 | 0.00037 |
| 3 | Average | 0.0021 | 0.57 | $1.9 \times 10^{-11}$ | 0.87 | $4.6 \times 10^{-5}$ |
| | ET | $3.2 \times 10^{-19}$ | $5.1 \times 10^{-6}$ | $3.7 \times 10^{-17}$ | NA | $2.3 \times 10^{-22}$ |
| | TC | 0.35 | NA | $2.3 \times 10^{-6}$ | 0.37 | 0.76 |
| | WT | 0.026 | 0.00071 | NA | 0.24 | 0.88 |
| 4 | Average | 0.53 | $6.5 \times 10^{-8}$ | 0.16 | 0.0094 | 0.77 |
| | ET | $3.2 \times 10^{-19}$ | $5.1 \times 10^{-6}$ | $3.7 \times 10^{-17}$ | NA | $2.3 \times 10^{-22}$ |
| | TC | 0.35 | NA | $2.3 \times 10^{-6}$ | 0.37 | 0.76 |
| | WT | $4.0 \times 10^{-26}$ | $7.7 \times 10^{-21}$ | $3.7 \times 10^{-24}$ | $4.9 \times 10^{-10}$ | $1.3 \times 10^{-12}$ |

Table 3: Demographic information of all cases used in this study.

| **Site ID** | **Total Cases** | **SEX** | | | **AGE: Male** | | | | **AGE: Female** | | | | **IDH status** | | |
|---|---|---|---|---|---|---|---|---|---|---|---|---|---|---|---|
| | | **M** | **F** | **NA** | **Average** | **Std.Dev** | **Min** | **Max** | **Average** | **Std.Dev** | **Min** | **Max** | **mutant** | **wildtype** | **NOS** |
| 1 | 827 | 495 | 332 | 0 | 59.93 | 14.57 | 10 | 89 | 61.22 | 13.55 | 20 | 86 | 96 | 482 | 249 |
| 2 | 611 | 367 | 244 | 0 | 62.32 | 12.43 | 18.65 | 87.59 | 64.03 | 12.34 | 20.74 | 88.5 | 16 | 499 | 96 |
| 3 | 400 | 242 | 158 | 0 | 59.6 | 13.8 | 18 | 94 | 62.3 | 12.9 | 19 | 89 | 24 | 376 | 0 |
| 4 | 221 | 88 | 69 | 64 | 42.29 | 16.56 | NA | NA | 37.34 | 16.43 | NA | NA | 57 | 72 | 92 |
| 5 | 193 | 92 | 101 | 0 | 56.08 | 13.42 | 24 | 80 | 52.04 | 14.99 | 18 | 78 | 45 | 47 | 101 |
| 6 | 120 | 73 | 47 | 0 | 52.7 | 15.6 | 22 | 81 | 50.2 | 15.3 | 22 | 71 | 29 | 88 | 3 |
| 7 | 120 | 73 | 47 | 0 | 57.1 | 14.92 | 24 | 81 | 56.42 | 14.78 | 21 | 85 | 22 | 98 | 0 |
| 8 | 120 | 72 | 48 | 0 | 59.9 | 10.25 | 36 | 79 | 61.2 | 9.59 | 28 | 74 | 3 | 117 | 0 |
| 9 | 118 | 64 | 54 | 0 | 58.76 | 11.21 | 23 | 77 | 59.03 | 10.66 | 31 | 77 | 4 | 78 | 36 |
| 10 | 114 | 64 | 50 | 0 | 60.95 | 11.54 | 30 | 86 | 61.14 | 15 | 18 | 85 | 2 | 112 | 0 |
| 11 | 112 | 0 | 0 | 112 | NA | NA | NA | NA | NA | NA | NA | NA | 0 | 0 | 112 |
| 12 | 108 | 69 | 39 | 0 | 61.15 | NA | 18 | 88 | 60.15 | NA | 18 | 88 | 0 | 0 | 108 |
| 13 | 108 | 78 | 30 | 0 | 54.3 | 14.2 | 24 | 82 | 66 | 9.9 | 29 | 85 | 5 | 51 | 52 |
| 14 | 107 | 63 | 44 | 0 | 58.36 | 10.79 | 29.3 | 79.1 | 59.89 | 10.79 | 43.1 | 78.3 | 2 | 46 | 59 |
| 15 | 104 | 66 | 38 | 0 | 57.29 | 15.85 | 7 | 86 | 61.45 | 16.13 | 30 | 87 | 10 | 42 | 52 |
| 16 | 101 | 60 | 41 | 0 | 54.89 | 13.6 | 27 | 79 | 56.9 | 14.05 | 25 | 90 | 6 | 35 | 60 |
| 17 | 100 | 65 | 35 | 0 | 61.88 | NA | NA | NA | 63.44 | NA | NA | N/A | 0 | 0 | 100 |
| 18 | 100 | 54 | 46 | 0 | 58.22 | 12.25 | 27 | 77 | 55.93 | 16.83 | 19 | 78 | 12 | 88 | 0 |
| 19 | 100 | 69 | 30 | 1 | 60 | 12.5 | 25 | 81 | 61.7 | 12.8 | 36 | 87 | 11 | 88 | 1 |
| 20 | 100 | 62 | 38 | 0 | 64.31 | 11.82 | 31 | 87 | 61.08 | 14.41 | 22 | 84 | 9 | 83 | 8 |
| 21 | 100 | 72 | 28 | 0 | 53.4 | 15.7 | 18 | 84 | 52.8 | 15.9 | 20 | 84 | 22 | 78 | 0 |
| 22 | 94 | 33 | 34 | 27 | 56.3 | 12.4 | 29.17 | 79.31 | 60.13 | 15.09 | 23.27 | 83.66 | 7 | 56 | 31 |
| 23 | 93 | 51 | 42 | 0 | 51.05 | NA | NA | NA | 57 | NA | NA | NA | 15 | 78 | 0 |
| 24 | 90 | 47 | 43 | 0 | 55 | 16 | 13 | 86 | 62 | 11 | 28 | 81 | 5 | 85 | 0 |
| 25 | 89 | 47 | 42 | 0 | 61.45 | 11.73 | 34 | 80 | 65.97 | 10.56 | 23 | 79 | 9 | 27 | 53 |
| 26 | 85 | 0 | 0 | 85 | NA | NA | NA | NA | NA | NA | NA | NA | 0 | 0 | 85 |
| 27 | 85 | 58 | 27 | 0 | 63.36 | 10.49 | 34 | 83 | 59.81 | 10.69 | 29 | 82 | 5 | 53 | 27 |
| 28 | 84 | 42 | 42 | 0 | 61.03 | 13.64 | 12.08 | 87.78 | 63.35 | 13.51 | 12.13 | 82.08 | 3 | 81 | 0 |
| 29 | 81 | 52 | 29 | 0 | 59.3 | NA | NA | NA | 58.2 | NA | NA | NA | 3 | 51 | 27 |
| 30 | 81 | 47 | 34 | 0 | 62 | 12 | 16 | 84 | 59 | 11 | 33 | 80 | 0 | 0 | 81 |
| 31 | 80 | 49 | 31 | 0 | 51.66 | 14.39 | 15 | 81 | 50 | 12.33 | 21 | 69 | 1 | 14 | 65 |
| 32 | 80 | 57 | 23 | 0 | 55.53 | 11.96 | 34 | 81 | 53.57 | 11.96 | 32 | 79 | 5 | 66 | 9 |
| 33 | 78 | 47 | 31 | 0 | 58.9 | 10.69 | 22.52 | 76.58 | 59.4 | 11.06 | 30.25 | 81.7 | 4 | 71 | 3 |
| 34 | 70 | 45 | 25 | 0 | NA | NA | NA | NA | NA | NA | NA | NA | 0 | 0 | 70 |
| 35 | 70 | 45 | 25 | 0 | 64.17 | 9.79 | 40 | 85 | 63.8 | 11.35 | 35 | 83 | 6 | 61 | 3 |
| 36 | 67 | 39 | 28 | 0 | 64.1 | 11.6 | 44 | 87 | 60.5 | 13.1 | 28 | 83 | 5 | 50 | 12 |
| 37 | 66 | 48 | 18 | 0 | 65.0 | 14.7 | 36 | 91 | 60.6 | 10.3 | 36 | 70 | 0 | 0 | 66 |
| 38 | 65 | 30 | 35 | 0 | 61.23 | 14.47 | 30 | 85 | 61.85 | 14.5 | 29 | 84 | 0 | 50 | 15 |
| 39 | 65 | 38 | 25 | 2 | 57.16 | 13.51 | 20 | 79 | 60.44 | 13.01 | 19 | 81 | 2 | 29 | 34 |
| 40 | 64 | 36 | 27 | 1 | 61.03 | 11.04 | 38 | 81 | 60.63 | 11.4 | 36 | 82 | 0 | 64 | 0 |
| 41 | 64 | 24 | 16 | 24 | NA | NA | NA | NA | NA | NA | NA | NA | 0 | 0 | 64 |
| 42 | 59 | 37 | 22 | 0 | 61.9 | 12.7 | 20 | 91 | 64.2 | 10.7 | 32 | 74 | 1 | 58 | 0 |
| 43 | 58 | 36 | 19 | 3 | 54.36 | 13.13 | 20 | 76 | 55.79 | 14.21 | 24 | 75 | 5 | 53 | 0 |
| 44 | 51 | 33 | 18 | 0 | 55.1 | 11.8 | 35 | 77 | 61.6 | 12.3 | 37 | 80 | 1 | 50 | 0 |
| 45 | 50 | 35 | 10 | 5 | NA | NA | NA | NA | NA | NA | NA | NA | 0 | 0 | 50 |
| 46 | 48 | 27 | 21 | 0 | 56.23 | 12.86 | 27 | 80.3 | 57.84 | 11.64 | 35 | 76.31 | 0 | 0 | 48 |
| 48 | 46 | 24 | 22 | 0 | 50.79 | 14.39 | 19 | 72 | 49.68 | 18.46 | 19 | 79 | 2 | 10 | 34 |
| 49 | 46 | 20 | 12 | 14 | 58.25 | 11.29 | 28 | 82 | 55.67 | 13.32 | 25 | 72 | 0 | 3 | 43 |
| 50 | 43 | 24 | 19 | 0 | NA | NA | 31 | 82 | NA | NA | 31 | 82 | 0 | 0 | 43 |
| 52 | 32 | 17 | 15 | 0 | 58.8 | 10.4 | 35 | 81 | 59.6 | 12.6 | 31 | 78 | 1 | 4 | 27 |
| 53 | 30 | 0 | 0 | 30 | NA | NA | NA | NA | NA | NA | NA | NA | 0 | 0 | 30 |
| 54 | 25 | 0 | 0 | 25 | 58.21 | 12.42 | 44.33 | 75.32 | 58.21 | 12.42 | 44.33 | 75.32 | 0 | 0 | 25 |
| 56 | 23 | 11 | 12 | 0 | 35.09 | 17.34 | 7 | 57 | 42.16 | 22.19 | 5 | 76 | 0 | 0 | 23 |
| 59 | 19 | 0 | 0 | 19 | NA | NA | NA | NA | NA | NA | NA | NA | 0 | 0 | 19 |
| 60 | 18 | 12 | 6 | 0 | 50 | 21 | 12 | 72 | 57 | 9 | 43 | 63 | 2 | 9 | 7 |
| PIM: 47, 51, 55, 57, 58, 61, 62, 63, 64, 65, 66, 67, 68, 69, 70, 71 | 231 | 116 | 92 | 23 | 57.02 | 14.37 | 17.47 | 81.21 | 53.22 | 14.08 | 17.7 | 84.84 | 0 | 0 | 231 |
